# The roles of autophagy, ferroptosis and pyroptosis in the anti-ovarian cancer mechanism of harmine and their crosstalk

**DOI:** 10.1038/s41598-024-57196-7

**Published:** 2024-03-18

**Authors:** Jun Zhu, Hong Zhu, Qing Zhu, Shi Lei Xu, Lu Xiao, Ming Yue Zhang, Jun Gao

**Affiliations:** 1 Jiangxi Medical College, Nanchang University, Nanchang, 330036 Jiangxi China; 2 Jiangxi Provincial Key Laboratory of Tumor Metastasis and Precision Therapy, Nanchang, Jiangxi China; 3 Department of Gynecologic Oncology, Jiangxi Cancer Hospital, Nanchang, Jiangxi China; 4 Nanchang Key Laboratory of Precision Therapy for Gynecological Oncology, Nanchang, Jiangxi China; 5 The 334 Hospital of Nanchang, Nanchang, Jiangxi China; 6 Jiangxi University of Chinese Medicine, Nanchang, Jiangxi China

**Keywords:** Ovarian cancer, Drug delivery, Autophagy, Cell death

## Abstract

This study aimed to investigate the role of autophagy, ferroptosis, and pyroptosis in the antitumour mechanism of harmine (Har) and its crosstalk in ovarian cancer. By transmission electron microscopy, we found that compared with those in the control group, the cytoplasm of human ovarian cancer cells (SKOV3) treated with Har showed increased numbers of autophagic vesicles, decreased intracellular mitochondrial volume, increased bilayer membrane density, and decreased cristae. Western blot, immunofluorescence, and monodasylcadaverine (MDC) staining all suggested that Har promoted autophagy in SKOV3 cells. LY294002 and siFOXO3 rescued the inhibition of the PI3K/AKT/mTOR/FOXO3 signalling pathway and the promotion of autophagy by Har. Additionally, the levels of ferroptosis- and pyroptosis-related proteins and the levels of Fe^2+^ , glutathione (GSH), malondialdehyde (MDA), and superoxide dismutase (SOD) suggested that Har promoted ferroptosis and pyroptosis in SKOV3 cells. Interestingly, pretreatment with chloroquine (CQ), erastin, rapamycin (Rap), or ferrostatin-1 (Fer-1) increased or reversed the ferroptosis and pyroptosis promoted by Har, respectively. In vivo, the volume of tumours in the Har group was decreased, and immunohistochemistry revealed decreased levels of Ki-67 and GPX4 and increased levels of ATG5 and NARL3. In conclusion, Har exerts its anti-ovarian cancer effect not only by promoting autophagy by regulating the PI3K/AKT/mTOR/FOXO3 signalling pathway but also by promoting ferroptosis and pyroptosis. Additionally, there is complex crosstalk between autophagy, ferroptosis, and pyroptosis in ovarian cancer.

## Introduction

Ovarian cancer, the most fatal malignancy of the female reproductive system, seriously threatens women's health. Resistance and severe side effects limit the efficacy of chemotherapy treatment in patients with ovarian cancer, and therefore impact the prognosis of these patients. As a result, finding effective antitumour medicines has become an urgent meed. Har is a natural beta-caroline alkaloid isolated from camel seeds^1^, that has insecticidal, antibacterial, anti-inflammatory, analgesic, antiviral, antidepressant and antitumor effects^2–4^.Several studies have demonstrated that Har can play an antitumour role in liver cancer, gastric cancer, colorectal cancer, non-small cell lung cancer, and breast cancer through various mechanisms^5–10^.

Our previous study suggested that Har can exert an anti-ovarian cancer effect by inhibiting proliferation and migration, but its specific mechanism is still unclear^10^. Currently, all non-surgical treatments for ovarian cancer are intended to induce tumor cell death. However, resistance to apoptosis affects the efficacy of antitumour treatment. With the extensive study of programmed cell death in malignant tumours, autophagy, ferroptosis and pyroptosis have been shown to play important roles in the genesis, development and outcome of tumours^11–13^. There is crosstalk between various cell death regulatory pathways, opening up new ideas for antitumour therapy^14^.

Autophagy is a double-edged sword in the development and progression of tumours. It can not only provide energy for cancer cells but also induce the autophagic death of tumour cells^15^. Ferroptosis appears as membrane damage resulting from the accumulation of iron and cellular lipid peroxidation^16^. It has also been proposed that the induction of autophagy-dependent ferroptosis may be a novel antitumour strategy^17^. Pyroptosis is a highly inflammatory form of programmed cell death that is triggered by inflammasome activation and mediated by gasdermin family proteins^18^. Currently, there is a lack of related studies on ferroptosis and pyroptosis, which are involved in the antitumour mechanisms of Har, and there are few studies on the crosstalk between autophagy, ferroptosis, and pyroptosis. To further explore the mechanism by which Har protects against ovarian cancer and lay a theoretical foundation for the clinical use of Har in ovarian cancer treatment, we investigated the role of autophagy, ferroptosis and pyroptosis in the antitumour mechanism of Har and the crosstalk between these processes in ovarian cancer in vivo and in vitro.

## Results

### Har induces autophagy in SKOV3 cells

Overall, we conducted a CCK-8 assay on multiple ovarian cancer cell lines SKOV3, A2780 and ES2 cells to verify the inhibitory effect of Har on ovarian cancer (Fig. S0a). We selected SKOV3 cells and Har (20, 30 μM) for subsequent experiments. In addition, we also verified the safety of the doses we used by detecting the effect of Har on the noncancer cell lines ISOE80 and 293 T cells. The results show that Har (20, 30 μM) was more toxic to SKOV3, A2780 and ES2 cells than to the noncancer cell lines ISOE80 and 293 T cells (Fig. S0b). This finding verified the safety of the doses of Har we used in this study.

**Figure 1:**
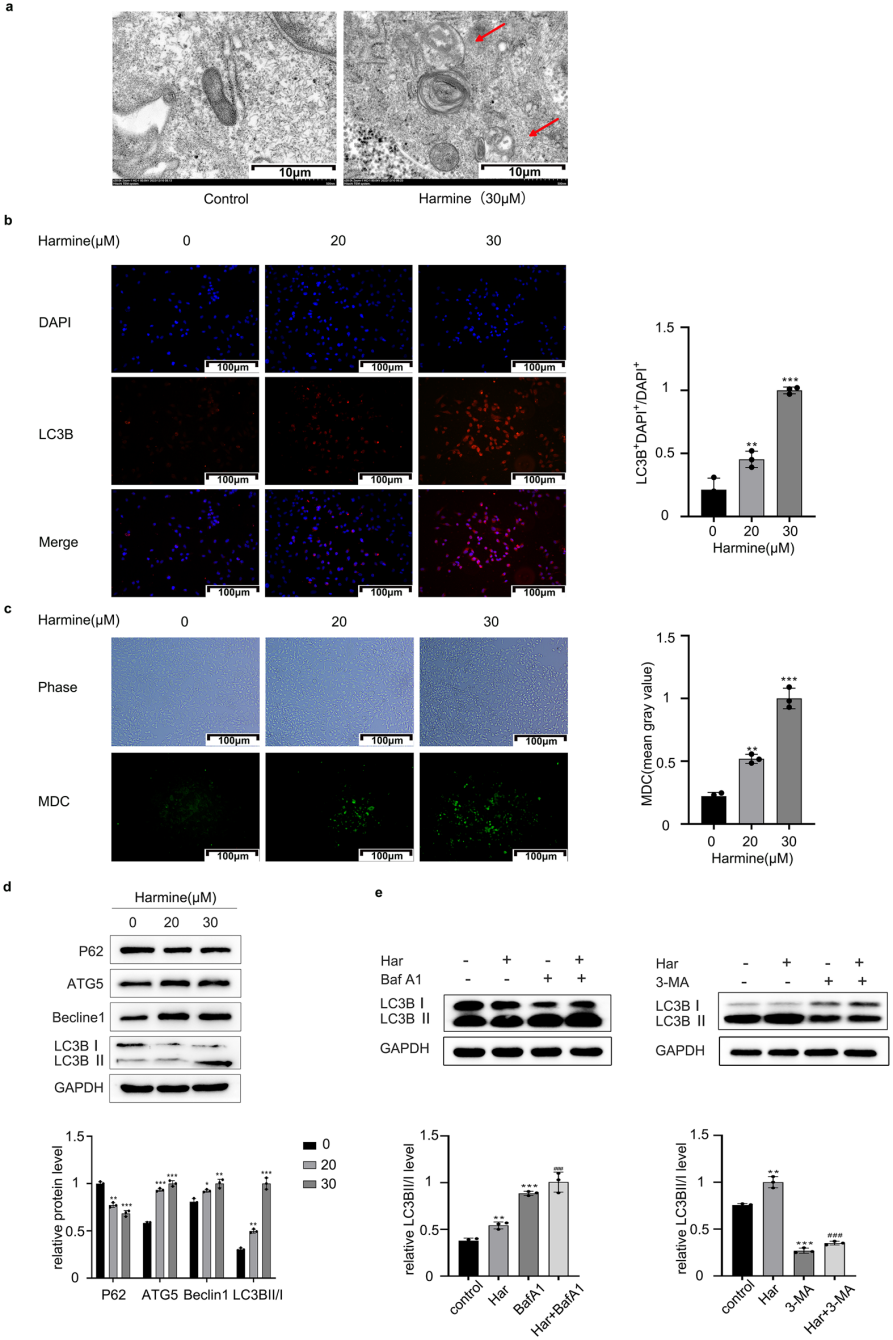
Har induced autophagy in SKOV3 cells. (**a**) Effects of Har on ultrastructural features of autolysosomes in SKOV3 cells (scale bar = 10 μm). (**b**) Fluorescence microscopy (scale bar = 100 μm) was used to observe the accumulation of LC3B-stained autophagic in control and Har-treated (0, 20, and 30 μM) SKOV3 cells. (**c**) Fluorescence microscopy (scale bar = 100 μm)) was used to observe the accumulation of MDC-stained cells of Har treated (0, 20, and 30 μM). (**d**) Protein expression of LC3B, ATG5, Beclin1 and P62 of SKOV3 cells treated with Har (0, 20, and 30 μM). (**e**) Protein expression of LC3B in SKOV3 cells treated with Har alone, or in combination with BafA1 (100 nM) or 3-MA (5 mM). GAPDH was used as an internal standard. The full-length original images (cropped) of representative blots are presented in Supplementary Fig. S1d and S1e of the Supplementary Information file. All results are expressed as the x ± s. *p < 0.05, **p < 0.01, ***p < 0.001 vs. the control group. #*p* < 0.05, ##*p* < 0.01, ###*p* < 0.001 vs. the Har group. *Har* harmine, *BafA1* bafilomycinA1, *3-MA* 3-Methyladenine.

Next, to explore the regulation of autophagy by Har, we first visualized the ultrastructure of autophagic vacuoles in SKOV3 cells via TEM. As shown in Fig. 1a, autopholysome (monolayer, and the cytoplasmic components was degraded) were present in the cytoplasm of SKOV3 cells treated with Har (30 μM), while the nuclei appeard normal. Autopholysomes were rarely observed in untreated cells. Then, we evaluated the intracellular level of autophagy in SKOV3 cells after treatment with Har (0, 20, 30 μM) for 48 h by performing LC3B immunofluorescence (Fig. 1b) and MDC staining (Fig. 1c). As depicted in Fig. 1b, the LC3B fluorescence(red) was weak in the control group, while significantly enhanced in the Har group. Additionally, Har treatment led to the accumulation of MDC-stained autophagic vacuoles (Fig. 1c).

We also examined the protein levels of autophagy-related markers. Among them, LC3-II, ATG5 and Beclin1 are essential for autophagosome formation, while p62 serves as an autophagy-specific substrate that is inversely associated with autophagic activity. As shown in Fig. 1d, compared with those in the control group, the protein levels of LC3-II/I, ATG5 and Beclin1 were increased, while the protein level of p62 was downregulated in cells decreased with Har for 48 h. To further assess autophagic flux, we cells with Har along with BafA1 or 3-MA. After pretreatment with BafA1, the degradation of autophagic lysosomes was inhibited, leading to the accumulation of LC3BII. Therefore, the change in LC3BII caused by Har only represents the change in autophagosome. As shown in Fig. 1e, the LC3BII levels in the Har + BafA1 group and Har + 3-MA group were greater than those in the BafA1 group and 3-MA group, respectively, indicating that Har can increase the synthesis of autophagosomes. In summary, Har promoted autophagy in SKOV3 cells.

### Har induced autophagy by inhibiting the PI3K/Akt/mTOR/FOXO3 signalling pathway

**Figure 2:**
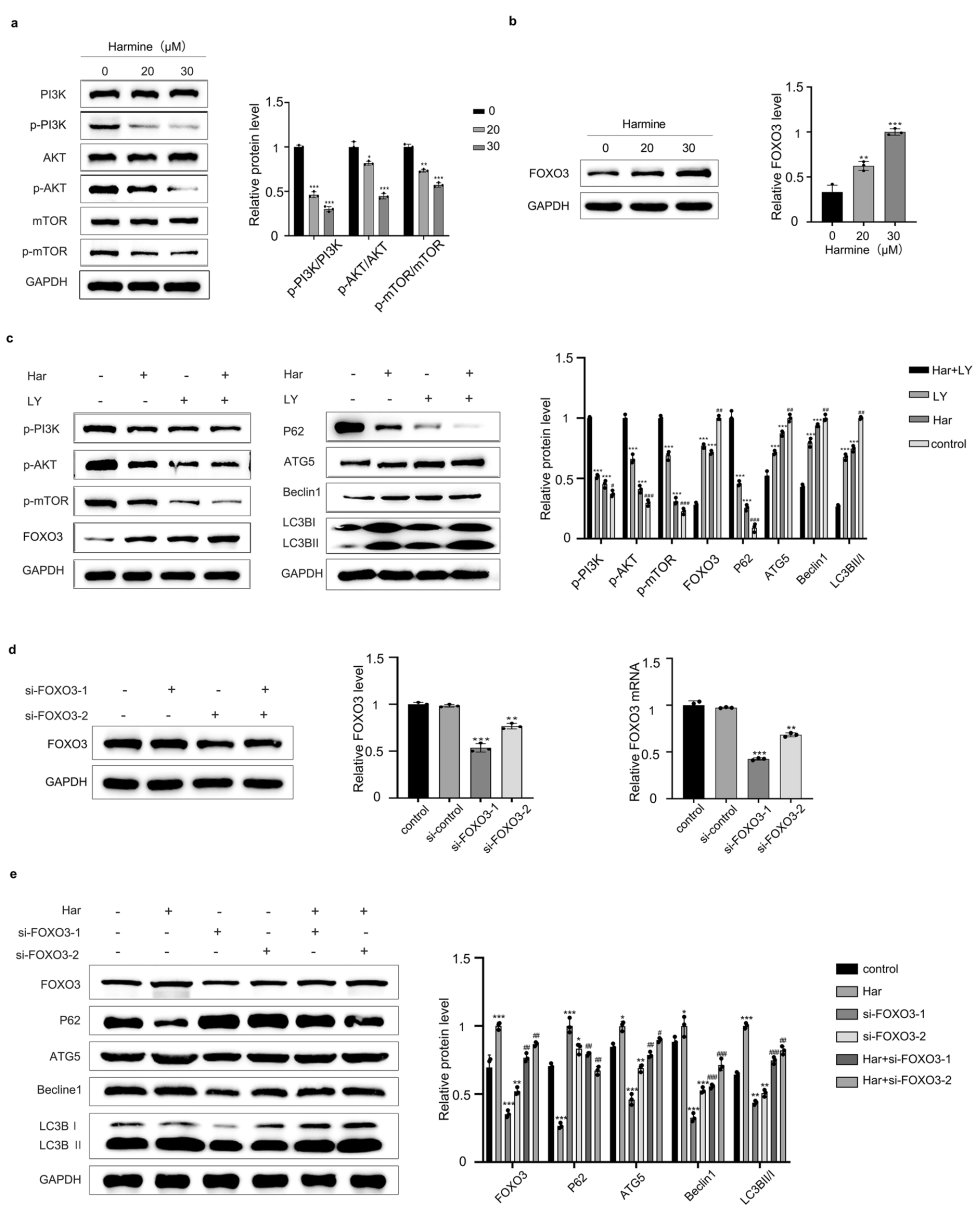
Har induced autophagy by inhibiting the PI3K/Akt/mTOR/FOXO3 signalling pathway. (**a**) Protein expression of PI3K, Akt, and mTOR and their phosphorylation levels in SKOV3 cells treated with Har (0, 20, and 30 μM). (**b**) Protein expression of P-PI3K, p- Akt, p-mTOR, FOXO3, and autophagy associated proteins in SKOV3 cells treated with Har (0, 20, and 30 μM). (**c**) FOXO3 protein expression in SKOV3 cells treated with Har andtreated with or without LY (a PI3K inhibitor). (**d**) The knockdown efficiency of siFOXO3 at the RNA and protein levels. (**e**) Protein expression of FOXO3, and autophagy associated proteins in SKOV3 cells treated with Har pretreatment with or without siFOXO3-1 or siFOXO3-2. GAPDH was used as an internal standard. The full-length original images (cropped) of representative blots are presented in Supplementary Fig. S2a, S2b, S2c, S2d, and S2e of the Supplementary Information file. All results are expressed as the x ± s. * p < 0.05, ** p < 0.01, *** p < 0.001 vs. the control group. #*p* < 0.05, ##*p* < 0.01, ###*p* < 0.001 vs. the Har group. *Har* harmine, *LY* LY294002.

To further explore the molecular mechanisms by which Har promotes autophagy in SKOV3 cells, we evaluated whether the classical PI3K / AKT / mTOR signalling pathway is involved in this process. As shown in Fig. 2a, treatment with Har (0, 20, 30 μM) for 48 h reduced the protein levells of p-mTOR, p-PI3K and p-AKT without affecting the total protein levels of mTOR, PI3K and AKT. The downstream transcription factors of mTOR include key molecules such as HIF-1α, c-Myc, and FOXO3. Among them, FOXO3 regulates the transcription of autophagy-related genes and can induce autophagy by upregulating the expression of ATGs and promoting the conversion of LC3-I to LC3-II. As shown in Fig. 2b, Har treatment increased the protein level of FOXO3. Pretreatment with LY (an inhibitor of PI3K) further enhanced the increase in autophagy and Foxo 3 protein levels induced by Har (Fig. 2c). To further demonstrate that FOXO3 is involved in the regulation of autophagy by Har in SKOV3 cells, cells were treated with Har after FOXO3 was knocked down. As shown in Fig. 2e, the levels of autophagy in both the Har + siFOXO3-1 group and the Har + siFOXO3-2 group were lower than those in the Har group, indicating that knockdown of FOXO3 attenuated the effect of Har on promoting autophagy in SKOV3 cells. Figure 2d shows the knockdown efficiency of siFOXO3-1 and siFOXO3-2.

### Har induced ferroptosis in SKOV3 cells

**Figure 3:**
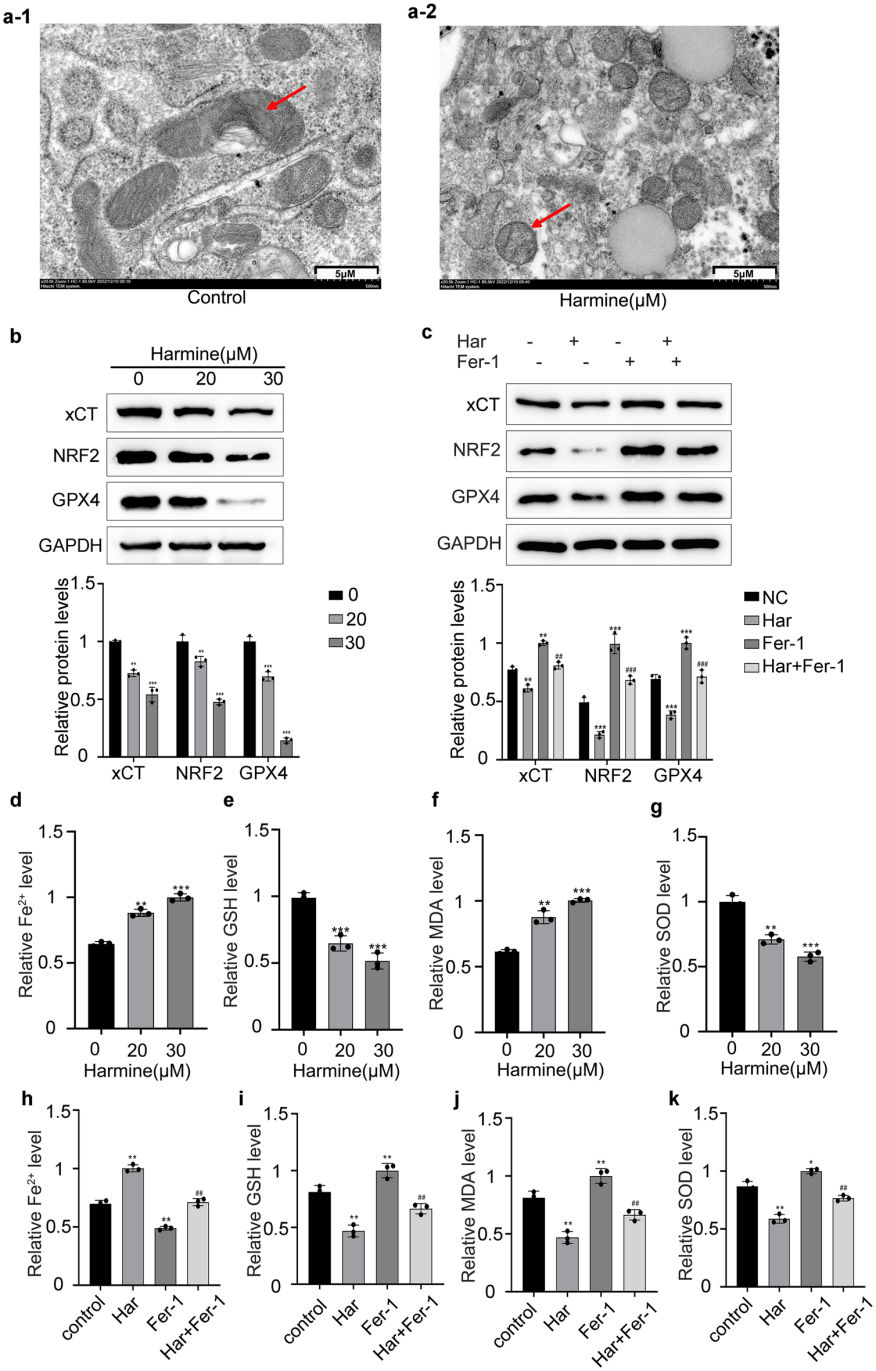
Har induced ferroptosis in SKOV3 cells. (**a**) Effects of Har on ultrastructural features of mitochondrion in SKOV3 cells (scale bar = 5 μM). (**b**) Protein expression of xCT/SLC7A11, NRF2, and GPX4 of SKOV3 cells treated with Har (0, 20, and 30 μM). (**c**) Protein expression of xCT/SLC7A11, NRF2, and GPX4 of SKOV3 cells treated with Har and pretreatment with or without Fer-1 (ferroptosis inhibitor). GAPDH was used as an internal standard. (**d**–**k**) Effects of Har and Fer-1 on iron, GSH, MDA, SOD, and content in SKOV3 cells. The full-length original images (cropped) of representative blots were presented in the supplementary Fig. S3b, S3c of Supplementary information file. All results are expressed as the x ± s. *p < 0.05, **p < 0.01, ***p < 0.001 vs. the control group. #*p* < 0.05, ##*p* < 0.01, ###*p* < 0.001 vs. the Har group. *Har* Harmine, *Fer-1* ferrostatin-1.

To explore the regulation of ferroptosis by Har, we first visualized the mitochondrial ultrastructure via TEM. As shown in Fig. 3a, SKOV3 cells treated with Har (30 μM) had a decreased intracellular mitochondrial volume, increased bilayer membrane density, and decreased cristae in the cytoplasm (Fig. 3a-2) compared with those in the control group (Fig. 3a-1).

In addition, we detected the protein levels of negative regulators of ferroptosis. As shown in Fig. 3b, Har downregulated the protein levels of xCT/SLC7A11, NRF2, and GPX4. Finally, the accumulation of Fe^2+^ and MDA, as well as the depletion of GSH and SOD, which are antioxidants, are typical signs of ferroptosis. Therefore, we also detected that Har not only upregulate the contents of Fe^2+^ and MDA, but also downregulate the contents of GSH and SOD (Fig. 3d–g). Moreover, the above indicators in the Fer-1 + Har group were intermediate between those in Har group, and the Fer-1 group (Fig. 3c,h–k). Therefore, Fer-1 reversed the promotion of ferroptosis by Har. Taken together, these findings indicate that Har can induce ferroptosis in SKOV3 cells.

### Inhibition of autophagy attenuate the ferroptosis induced by Har in SKOV3 cells

**Figure 4:**
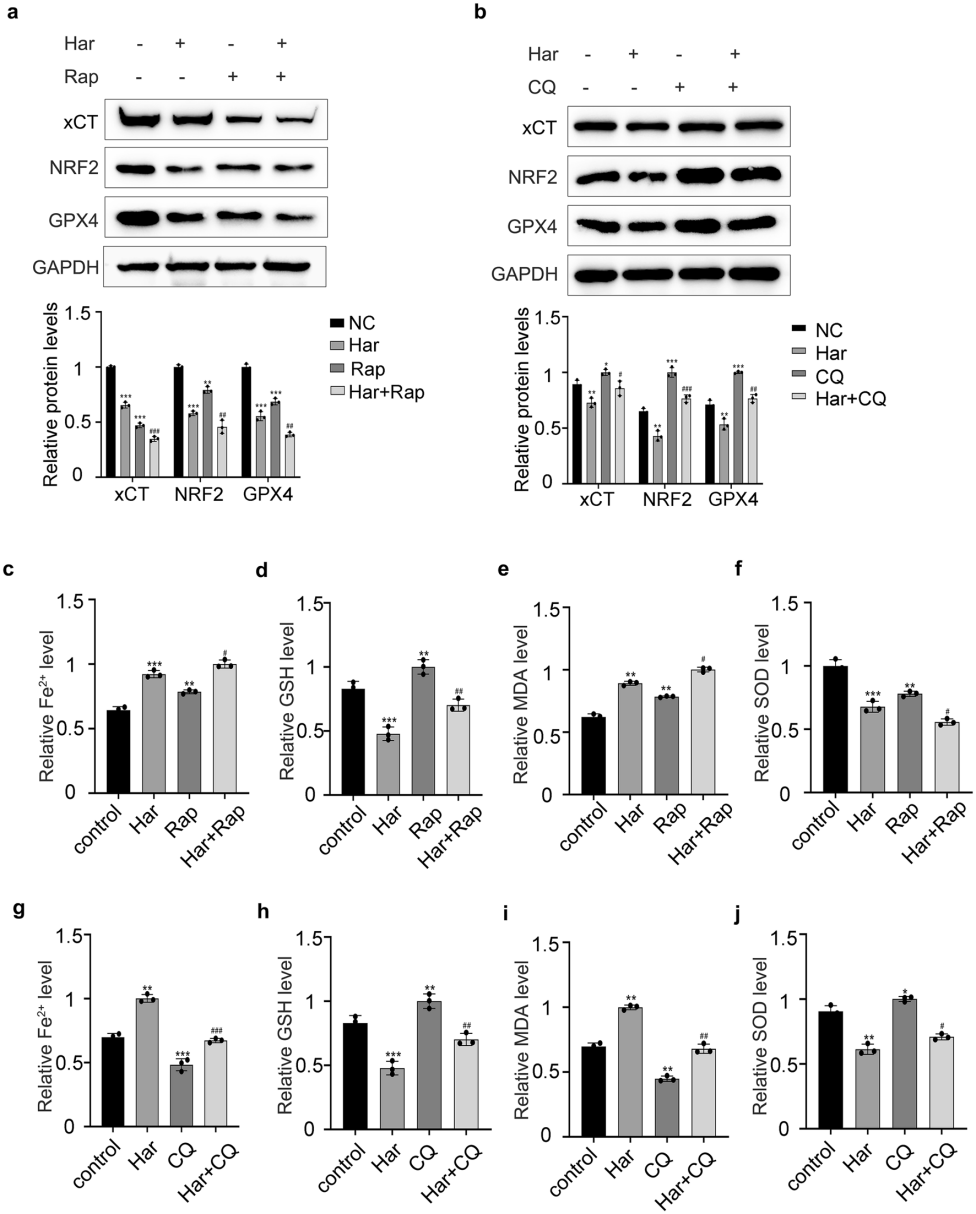
Inhibition of autophagy attenuate the ferroptosis induced by Har in SKOV3 cells. (**a**) The effect of Rap on protein levels of xCT/SLC7A11, NRF2, and GPX4 in Har-treated SKOV3 cells. (**b**) The effect of CQ on protein levels of xCT/SLC7A11, NRF2, and GPX4 in Har-treated SKOV3 cells. GAPDH was used as an internal standard. (**c**–**f**) Effects of Har and Rap on iron, GSH, MDA, SOD, and content in SKOV3 cells. (**g**–**j**) Effects of Har and CQ on iron, GSH, MDA, SOD, and content in SKOV3 cells. The full-length original images (cropped) of representative blots were presented in the supplementary Fig. S4a, S4b of Supplementary information file. All results are expressed as the mean ± SD. All results are expressed as the x ± s. *p < 0.05, **p < 0.01, ***p < 0.001 vs. the control group. #*p* < 0.05, ##*p* < 0.01, ###*p* < 0.001 vs. the Har group. *Har* Harmine, *Rap* Rapamycin, *CQ* chloroquine.

We tested the changes in ferroptosis levels after treating SKOV3 cells with Rap and CQ alone or in combination with Har. As shown in Fig. 4a, similar to those in the Har group, the protein levels of xCT/SLC7A11, NRF2 and GPX4 as well as those of GSH (Fig. 4d) and SOD (Fig. 4f), and upregulated the levels of Fe^2+^ (Fig. 4c) and MDA (Fig. 4e). In contrast, CQ had the opposite effect (Fig. 4b,g–j). In addition, compared to that in the Har group, Rap further promoted ferroptosis induced by Har, while CQ alleviated ferroptosis induced by Har in SKOV3 cells. In conclusion, autophagy is involved in Har-induced ferroptosis.

### Both autophagy and ferroptosis participate the anti-ovarian cancer mechanism of Har

**Figure 5:**
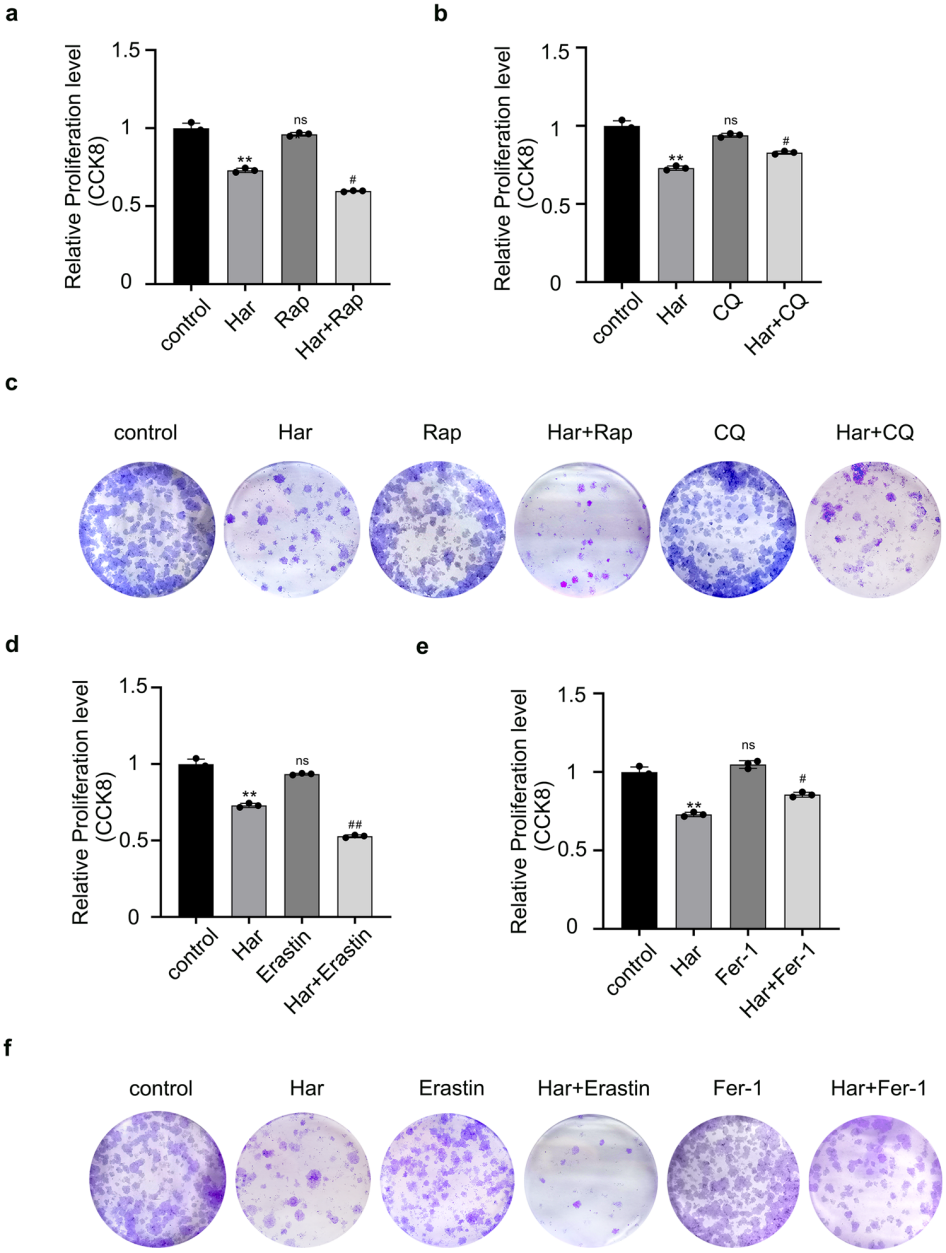
Both autophagy and ferroptosis participate the anti-ovarian cancer mechanism of Har. (**a**) Effects of Har on Rap-induced proliferation capacity of SKOV3 cells. (**b**) Effects of Har on CQ-induced proliferation capacity of SKOV3 cells. (**c**) Effects of Har on Rap or CQ pretreated colony formation capacity of SKOV3 cells. (**d**) Effects of Har on Erastin-induced proliferation capacity of SKOV3 cells. (**e**) Effects of Har on Fer-1-induced proliferation capacity of SKOV3 cells. (**f**)Effects of Har on Erastin or Fer-1 pretreated colony formation capacity of SKOV3 cells. All results are expressed as the x ± s. *p < 0.05, **p < 0.01, ***p < 0.001 vs. the control group. #*p* < 0.05, ##*p* < 0.01 vs. the Har group. *Har* Harmine, *Rap* Rapamycin, *CQ* chloroquine, *Fer-1* ferrostatin-1.

To further explore whether autophagy or ferroptosis is involved in the antitumour effect of Har, we conducted CCK-8 assays and clone formation assays to detect the proliferation of SKOV3 cells. As shown in Fig. 5a,b, pretreatment with both Rap and erastin further inhibited the proliferation of SKOV3 cells (Fig. 5a,c,d,f), while both CQ (an inhibitor of autophagy) and Fer-1(an inhibitor of ferroptosis) inhibited the proliferation of SKOV3 cells via Har(Fig. 5b,c,e,f). The above results indicated that both autophagy and ferroptosis were involved in the mechanism by which Har suppresses SKOV3 cell proliferation. In other words, Har can exert anti-ovarian cancer effects by promoting autophagy and ferroptosis in SKOV3 cells.

### Har-mediated autophagy and ferroptosis are involved in the induction of pyroptosis in SKOV3 cells

**Figure 6:**
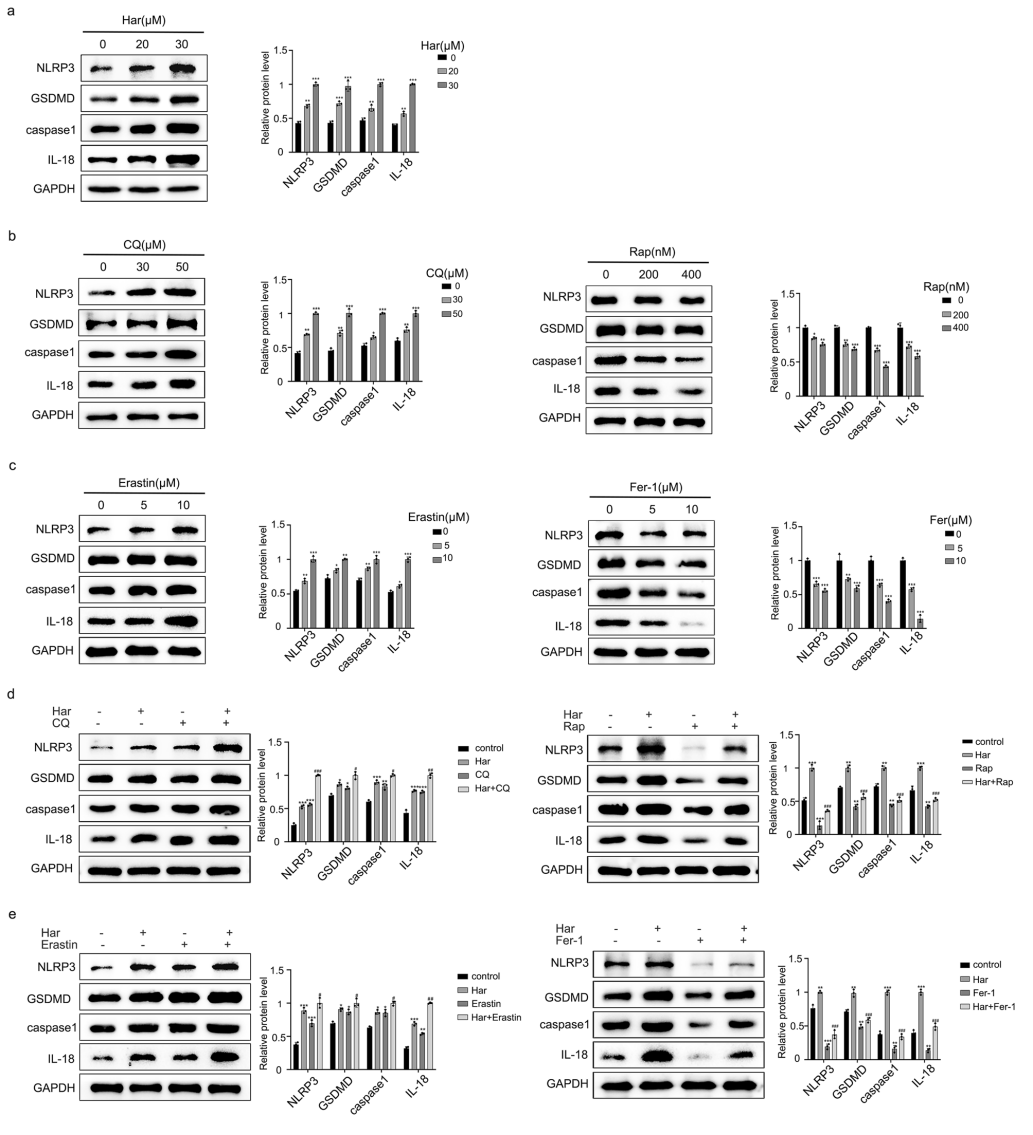
Autophagy and ferroptosis are involved in the induction of pyroptosis in SKOV3 cells by Har. (**a**) Protein expression of NARL3, casepase-1, IL-18, and GSDMD in SKOV3 cells treated with Har (0, 20, and 30 μM). (**b**) Protein expression of NARL3, casepase-1, IL-18, and GSDMD in SKOV3 cells treated with CQ (0, 20, and 30 μM) or Rap (0, 200, and 400 nM). (**c**) Protein expression of NARL3, casepase-1, IL-18, and GSDMD in SKOV3 cells treated with Eratin (0, 5, and 10 μM) and Fer-1 (0, 5, and 10 μM). (**d**) Effect of CQ and Rap on the protein levels of NARL3, casepase-1, IL-18, and GSDMD in Har-treated SKOV3 cells. (**e**) Effect of erastin and Fer-1 on the protein levels of NARL3, casepase-1, IL-18, and GSDMD in Har-treated SKOV3 cells. GAPDH was used as an internal standard. The full-length original images (cropped) of representative blots are presented in Supplementary Fig. S6a, S6b, S6c, S6d, and S6e of the Supplementary Information file. All results are expressed as the x ± s. *p < 0.05, **p < 0.01, ***p < 0.001 vs. the control group. #*p* < 0.05, ##*p* < 0.01, ###*p* < 0.001 vs. the Har group. *Har* harmine, *Rap* Rapamycin, *CQ* chloroquine, *Fer-1* ferrostatin-1.

An increase in NLRP3, caspase-1, GSDMD, and the release of IL-18 are the key signals of pyroptosis. As shown in Fig. 6a, Har upregulated the protein levels of NARL3, casepase-1, IL-18 and GSDMD. These findings suggest that Har induces pyroptosis in SKOV3 cells. To explore the crosstalk between autophagy, ferroptosis and pyroptosis, we detected changes in pyroptosis-related protein levels after regulating the levels of autophagy and ferroptosis with Rap, CQ, Fer-1, and erastin. As shown in Fig. 6b,c both Rap and Fer-1 downregulated the levels of pyroptosis-related proteins, while CQ, and erastin upregulated them. Moreover, pretreatment with CQ, Erastin and Rap, Fer-1 respectively increased and reversed the inhibition of pyroptosis by Har (Fig. 6d,e). In conclusion, autophagy and ferroptosis are involved in the induction of pyroptosis by Har.

### Har inhibited ovarian cancer cells in transplanted tumors

**Figure 7:**
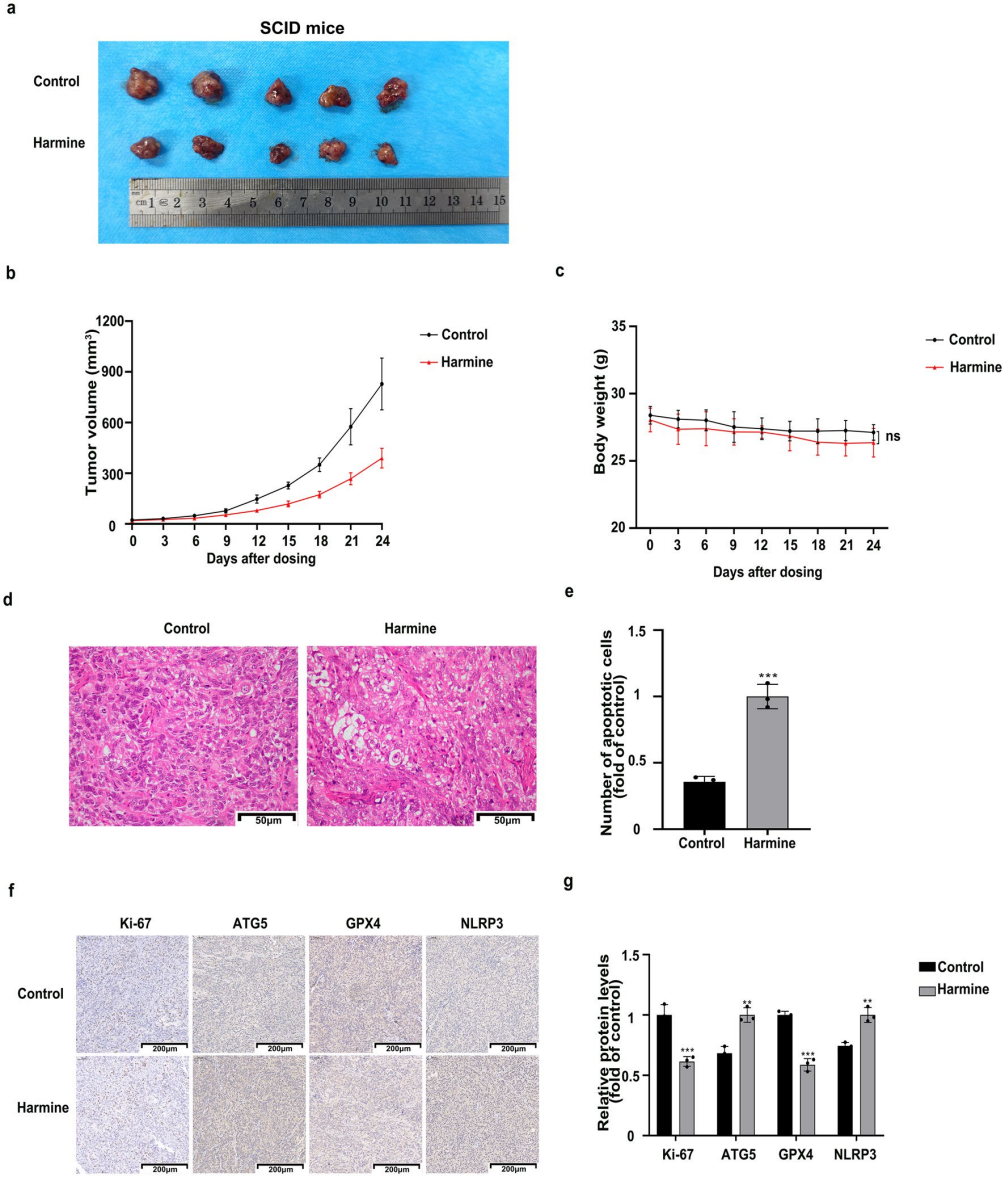
Har inhibited ovarian cancer in vivo. (**a**) Photo images of comparison of tumor masses size between the two groups. (**b**) The changes of body weight of mice between the two groups. (**c**) The changes of tumor volume between the two groups. (**d**, **e**) We observed the effect of Har on the morphology of ovarian cancer cells by HE stains. (**f**, **g**) The expression of Ki-67, ATG5, GPX4, and NLRP3 in tumor tissues between the two groups. All results are expressed as the x ± s. *p < 0.05, **p < 0.01, ***p < 0.001 vs. the control group. #*p* < 0.05, ##*p* < 0.01, ###*p* < 0.001 vs. the Har group.

We demonstrated the roles of autophagy, ferroptosis and pyroptosis in the anti-ovarian cancer mechanism of Har in vivo. Therefore, we sought to validate our findings in vitro through in vivo experiments. We monitored changes in the tumour growth and body weight of the SCID mice in the control group and Har group every three days for 24 days. The body weight and tumor volume of each SCID mouse were evaluated during the intervention period. The tumor volume in the Har group was significantly lower than that in the control group (Fig. 7a,b). There was no statistically significant difference in body weight between the control and Har groups (Fig. 7c). We observed the effect of Har on the morphology of ovarian cancer cells by HE staining. As shown in Fig. 7d,e compared to the control group cells, the Har group cells exhibited slightly less density, variable sizes, and an increased number of apoptotic cells (characterized by nuclear condensation, deep staining, and detachment from neighbouring cells). We isolated the tumours and performed IHC analysis using ATG5, GPX4, NLRP3, and Ki-67 staining. IHC analysis indicated that the protein level of ATG5 was higher and the level of GPX4, NLRP3, and Ki-67 was lower in the Har group than that in the control group (Fig. 7f,g). In conclusion, Har obviously inhibited the proliferation of ovarian cancer cells in transplanted tumors and promoted autophagy, ferroptosis and pyroptosis.

## Discussion

Nonapoptotic programmed cell death has been shown to play a very important role in tumorigenesis, development, and treatment. Previous studies have shown that Har can exert anti-ovarian cancer effects by inhibiting ovarian cancer cell proliferation. Therefore, this study was carried out to explore the role of autophagy, ferroptosis, and pyroptosis in the antitumour mechanism of Har and its crosstalk in ovarian cancer. Previous studies have shown that Har promotes autophagy in gastric cancer cells and melanoma cells by inhibiting the Akt/mTOR/p70S6K pathway to exert antitumour effects^19,20^. However, no study has investigated the role of autophagy in the mechanism by which Har protects against ovarian cancer. In this study, we found that Har upregulated the autophagy levels in SKOV3 cells, as evidenced by electron microscopy, LC3B immunofluorescence staining, MDC, and western blotting. Meanwhile, upregulation of LCBII / I and downregulation of P62 suggested that the autophagy of SKOV3 cells was enhanced and that the autophagic flow was unobstructed after treatment with Har. Moreover, pretreatment with BafA1 and 3-MA promoted the Har-induced increase in autophagy in SKOV3 cells. We also detected that the PI3K / AKT / mTOR /Foxo3 signalling pathway was involved in the regulation of autophagy in SKOV3 cells by Har.

Many studies have shown that ferroptosis plays an important role in the antitumour mechanisms^21^, and ferroptosis is expected to be a new target for ovarian cancer treatment^22^. For example, Mao G et al. reported that sodium molybdate can inhibit the growth of ovarian cancer cells by inducing ferroptosis^23^. However, there are no reports on the regulation of ferroptosis by Har. In this study, Har was found to promote ferroptosis, and Fer-1 could reverse the activation of ferroptosis and the inhibition of SKOV3 cells proliferation induced by Har. In conclusion, Har could inhibit the proliferation of SKOV3 cells by promoting ferroptosis, but the specific mechanism involved remains to be investigated.

Similarly, pyroptosis is also thought to be closely related to tumour occurrence, development and outcome. Tan et al. showed that the lncRNA HOTTIP is upregulated in ovarian cancer tissues and cell lines, and its knockdown can induce pyroptosis and thus hinder the progression of ovarian cancer^24^. In addition, osthole, cinnamon and 2-α-NETA have been proved to exert anticancer activity through GSDMD-mediated proptosis^25,26^. Some authors suggested that pyroptosis may be subtly related to autophagy^27^. There is no report on Har regulating pyroptosis, and few studies exist on the regulatory relationship between pyroptosis and autophagy. Besides exploring the role of autophagy, ferroptosis and pyroptosis in the anti-ovarian cancer mechanism of Har, this study also explored the regulatory relationship between autophagy and ferroptosis and pyroptosis.

As research progresses, increasing evidence suggests that autophagy is involved in ferroptosis^28^. For example, Liu J et al. reported that autophagy can promote ferroptosis by regulating VDAC3 ubiquitination through FBXW7 in acute lymphoblastic leukemia^17^. Sun et al. also reported that the inhibition of autophagy attenuated oxidative stress and the degradation of GPX4 induced by Fin56 in bladder cancer^29^. However, there are no relevant studies on autophagy-regulated ferroptosis in ovarian cancer. Our study revealed that Har inhibited the proliferative capacity of SKOV3 cells by promoting autophagy and ferroptosis. Moreover, autophagy can positively regulate the level of ferroptosis. This result may stem from the upregulation of autophagy by Har, which promotes the degeneration and degradation of ferritin, leading to the excessive accumulation of Fe^2+^, ROS, and lipid peroxides, the inactivation of GPX4, and the consumption of SOD and GSH, thus accelerating the ferroptosis process. It may also be because Beclin1, which is involved in autophagosome formation, forms a complex with SLC7A11, thereby promoting lipid peroxidation and ferroptosis^30^. However, the specific underlying mechanism still needs to be further explored.

Our study revealed that CQ and erastin further promoted the pyroptosis induced by Har in SKOV3 cells, while Rap and Fer-1 alleviated it. Therefore, both autophagy and ferroptosis are involved in the regulation of pyroptosis by Har in SKOV3 cells. And autophagy can negatively regulate pyroptosis, while ferroptosis can positively regulate pyroptosis. So, why can autophagy and ferroptosis regulate the level of pyroptosis in SKOV3 cells? First, the activation of autophagy can promote the degradation of NLRP3 by autophagosomes. That is, autophagy can reduce overactivation of the NLRP3 inflammasome^31^. In hepatocellular carcinoma, oestradiol inhibits autophagy and promotes pyroptosis in HCC cells by activating the AMPK/mTOR signalling pathway^32^. In contrast, Rap induces autophagy by inhibiting the AMPK/mTOR signalling pathway and thereby alleviates pyroptosis in Leydig cells^27^.

Regarding the regulatory relationship between ferroptosis and pyroptosis, Professor Wu Qiao's group reported that Fe2 + and drugs that induce ROS elevation can induce pyroptosis via a Tom20-Bax-caspase-GSDME pathway, thus controlling tumour development^33^. Iron agents can also be used in combination with chemotherapy agents to induce pyroptosis in vivo. Bergsbaken et al. reported that CD8 + T cells, which are antitumour immune cells, can simultaneously promote these two types of cell death^34^. On the one hand, CD8 + T cells can induce pyroptosis. On the other hand, IFN-γ secreted by CD8 + T cells downregulates SLC7A11 and causes the accumulation of lipid ROS, thus inducing ferroptosis^35^.

## Conclusions

In summary, Har not only promotes autophagy by regulating the PI3K/AKT/mTOR/FOXO3 signalling pathway to play an anti-ovarian cancer role, but also promotes ferroptosis and pyroptosis in ovarian cancer. In addition, there is complex crosstalk between autophagy, ferroptosis, and pyroptosis. Further studies of the crosstalk between these processes would be beneficial for revealing the mechanism of tumor development and providing a reference for the prevention and treatment of diseases. This study provides new insights into the antitumor mechanism of Har and may contribute to the development of new antiovarian cancer drugs.

## Materials and methods

### Cell culture and treatment

The human normal ovarian cell line IOSE80 and SKOV3, ES2, and A2780 ovarian cancer cell lines cells were purchased from Procell (Shanghai, China). 293 T cells were donated by Jiangxi Provincial Key Laboratory of Tumor Metastasis and Precision Therapy. The cells were grown in McCoy’s 5A, DMEM or 1640 medium supplemented with 10% fetal bovine serum, 0.1 mg/ml streptomycin, and 100 U/ml penicillin at 37 °C in a humidified incubator with 5% CO_2_. Har, bafilomycin A1(BafA1), 3-Methyladenine(3-MA), LY294002(LY), rapamycin(Rap), chloroquine (CQ) and ferrostatin-1(Fer-1) were purchased from Med Chem Express (Shanghai, China) and dissolved in dimethyl sulphoxide (DMSO). The cells were cultured for the indicated time after diluting the drug to its final concentration in the medium.

### Measurement of cell viability

Cell viability was determined using CCK-8 assay kit. SKOV3 cells were seeded into 96-well plates at a density of 5000 cells per well. Cells were treated with or without drugs for 20 h, and three replicates were used for each group. Then, 10 µL CCK8 solution and 100 µL medium were added to each well, and the plates were incubated at 37℃ for 1 h. Finally, the absorbance of each well at 450 nm was measured using a microplate reader (EnSpire, PerkinElmer Company, USA).

### Immunofluorescence staining

After fixation and permeabilization, the cells in each group were incubated overnight with the primary antibody againstLC3B at 4 °C. The next day, after washing with PBS, the cells were incubated with Alexa Fluor 594 AffiniPure goat anti-rabbit IgG (H + L) (YEASEN Biotech) for 1 h at room temperature. The cell nucleus was then stained with DAPI. Finally, the dishes were observed and photographed under a fluorescence microscope (IX73, Olympus Company, Japan) (Amax 591 nm, Emax 614 nm) at 200 × magnification.

### Monodasylcadaverine (MDC) staining

MDC is a fluorescent stain that can be used to detect autophagosomes. Following the manufacturer’s instructions of the kit, cells were stained with MDC solution for 45 min at room temperature, and protected from light. After eluting the excess dye solution, the cells were observed and photographed under a fluorescence microscope (IX73, Olympus Company, Japan) (excitation filter wavelength: 355 nm, blocking filter wavelength: 512 nm). The number of autophagosomes (green) was quantified using ImageJ software.

### Transmission electron microscopy (TEM)

The ultrastructures of SKOV3 cells treated with or without 30 μM Har treatment were examined by TEM (HT7800/HT7700, HITACHI Company, Japan). Cell samples from each group were sequentially fixed with 2.5% glutaraldehyde and 1% osmic acid. Then, they were successively dehydrated with different concentrations of ethanol. After embedding and curing, the sections were cut into 70-nm sections and double-stained with 3% uranyl acetate-lead citrate, and micrographs were taken for qualitative description.

### RNA interference

Small interfering RNAs (siFOXO3-1, siFOXO3-2, and sicontrol) were synthesized from GenePharma (Shanghai, China) (Table 1). A total of 500 µLof Opti-MEM was mixed with either 5 µL of siRNA (20 µM) or 5 µL Lipofectamine RNAiMAX and then incubated for 15 min at room temperature. SKOV3 cells were seeded into 6 -well plates, transfected with the siRNA listed above for 48 h and then treated with or without Har.

**Table 1:** The sequences of the small interfering RNAs.

Gene (human)	Sense (5′-3′)	Antisense (5′-3′)
FOXO3-1	GACGAUGAUGCGCCUCUCUTT	AGAGAGGCGCAUCAUCGUCTT
FOXO3-2	AAUGUGACAUGGAGUCCAUUA	UAAUGGACUCCAUGUCACAUU
Negative control	UUCUCCGAACGUGUCACGUTT	ACGUGACACGUUCGGAGAATT

### Real time (RT)-PCR

Total RNA was extracted using TRIzol reagent, and then was reverse transcribed into cDNA using the Prime Script RT reagent Kit (Takara, RR036A). The expression levels of genes were measured using the SYBR ExScript qRT‒PCR Kit (TaKaRa) with GAPDH as an internal control. PCR amplification was performed under the following conditions: 95 °C for 5 min; 38 cycles of 95 °C for 30 s, 60 °C for 40 s, and 72 °C for 45 s in real-time PCR Instrument (CFX96, Bio-Rad, USA) . The relative expression levels of genes were analyzed using the 2^-△△Ct^ method. Gene-specific PCR primer pairs were synthesized by Genecreate (Wuhan, China; Table 2).

**Table 2:** Sequences of primers used for RT‒PCR in this study.

Gene (human)	Forward (5′–3′)	Reverse (5′–3′)
FOXO3	GCGTGCCCTACTTCAAGGATAAG	GACCCGCATGAATCGACTATG
GAPDH	CATGAGAAGTATGACAACAGCCT	AGTCCTTCCACGATACCAAAGT

### Western blot

Cells were harvested after the prescribed time of drug treatment to extract total protein. The protein concentration was measured using a BCA assay kit and aligned with the lysate. After separating the proteins using sodium dodecyl sulphate polyacrylamide gel electrophoresis (SDS-PAGE) (Bio-Rad, Hercules, CA, USA), the proteins were transferred to a polyvinylidene fluoride (PVDF) membrane. Subsequently, after exposure to confining liquid at room temperature for 2 h, the PVDF membranes were incubated overnight at 4 ˚C with different primary antibodies, including anti-GADPH, anti-PI3 Kinase p85 alpha Antibody, AKT1/2/3, anti-Phospho-PI3-kinase p85- alpha/ gamma, anti-p-Akt(Ser473), anti-mTOR, anti-p-mTOR, anti-ATG5, anti-Beclin-1, anti-Xct/SLC7A11, anti-GPX4, anti-NRF2, anti-IL-18, anti-GSDMD, anti-Caspase-1 as well as anti-NALRP3, all of which were purchased from Abmart (Shanghai, China). anti-LC3B, anti-P62, and anti-FOXO3 were purchased from Cell Signaling Technology (Beverly, MA, USA). On another day, those unbound primary antibodies were eluted with PBST and incubated with the IgG goat anti-rabbit/ mouse IgG for 1 h. After being washed with PBST again, the bands were detected using enhanced chemiluminescence (luminous liquid brand) (Tanon 5200, China) and quantified with ImageJ software.

### Determination of Iron, ROS, MDA, T-SOD, GSH and MDC levels

The iron assay kit (E-BC-K773-M), GSH kit (E-BC-K030-M), T-SOD kit (E-BC-K020-M), and MDA kit (E-BC-K028-M) were purchased from Elabscience (Wuhan, China). Following the manufacturer’s instructions, SKOV3 cells were harvested and centrifuged after ultrasonication. The supernatant was used as the test sample, and the content of iron, GSH, MDA, and the activity of SOD were measured using a microplate reader (EnSpire, PerkinElmer Company, USA) and normalized to the protein concentration or cell count.

### Animals and treatment

Female SCID mice (18 ± 2 g, 5–6 weeks old) were purchased from SPF Biotechnology. This study was conducted in accordance with the Declaration of Basal. All procedures were performed in accordance with the protocol approved by the Anburui Biological Experimental Animal Ethics Committee of Fujian (Licence no.: IACUC FJABR 2022042101) and conformed to the ARRIVE guidelines. Ten mice were injected with 5 × 10^6^ SKOV3 cells subcutaneously into the right flanks. After their tumour were observed, the mice were randomly divided into two groups, the control group and the Har group, and given saline or Har (60 mg/kg/day) by intraperitoneal injection for 24 days. The body weight and tumor volume of each mouse were measured every 3 days during the intervention period. Tumor growth was calculated using the following formula: ${\text{V}} = 0.52{\text{LW}}^{2} .$

Finally, all tumor-bearing mice were euthanized by cervical dislocation after isoflurane anaesthesia. The tumoUrs were extracted for measurement, photographed and further histopathological analysis. This study was approved by Anburui Biological Experimental Animal Ethics Committee of Fujian (licence number: IACUC FJABR 2,022,042,101) and conformed to the ARRIVE guidelines. The in vivo Har concentration was selected based on previous studies. For example, Ding Y et al. showed that different doses of Har (20, 40 or 80 mg/kg/day) reduced tumor volume after 4 weeks injection, and no mice experienced significant organ damage^9^. In addition, some in vivo studies have shown that Har (10–50 mg/kg/d) can alleviate liver, kidney, and myocardial damage caused by other factors and has neuroprotective effects^36–39^.

### Immunohistochemistry

The tumors were embedded in paraffin and sliced for immunohistochemistry. The slices were blocked with goat serum for 30 min and incubated with primary antibodies (including anti-Ki-67, ATG5, GPX4, andNALRP3) at 4℃ overnight. On the following day, after incubation with secondary antibody for 30 min, the slices were stained by 3,3 '- diaminobenzidine (DAB) for 15 min. Finally, the nuclei were restained with haematoxylin, and the slices were photographed with 100C × magnification (Olympus Corporation).

### Statistical analysis

All the data are expressed as the means ± standard deviations and were analysed using GraphPad Prism 8 software. ANOVA was performed to determine the statistical differences between groups. *p* < 0.05 was considered statistically significantto indicate statistical significance.

## Ethics approval

This study was conducted in accordance with the Declaration of Basal. All procedures were performedin accordance with the protocol approved by the Anburui Biological Experimental Animal Ethics Committee of Fujian (Licence no.: IACUC FJABR 2022042101) and conformed to the ARRIVE guidelines.

### Supplementary Information

Supplementary Information 1.

Supplementary Information 2.

Supplementary Information 3.

Supplementary Information 4.

Supplementary Information 5.

Supplementary Information 6.

Supplementary Information 7.

Supplementary Information 8.

Supplementary Information 9.

Supplementary Information 10.

Supplementary Information 11.

Supplementary Information 12.

Supplementary Information 13.

Supplementary Information 14.

Supplementary Information 15.

Supplementary Information 16.

Supplementary Information 17.

## Data Availability

The data that support the findings of this study are available on request from the corresponding author upon reasonable request.
