# Supplementary material for: The roles of autophagy, ferroptosis and pyroptosis in the anti-ovarian cancer mechanism of harmine and their crosstalk
Source: Sci Rep. 2024 Mar 18;14:6504. doi: 10.1038/s41598-024-57196-7 (PMC10948856; doi:10.1038/s41598-024-57196-7)
Supplement: Supplementary file 1 — Supplementary Information 1. [file 41598_2024_57196_MOESM1_ESM.pdf]

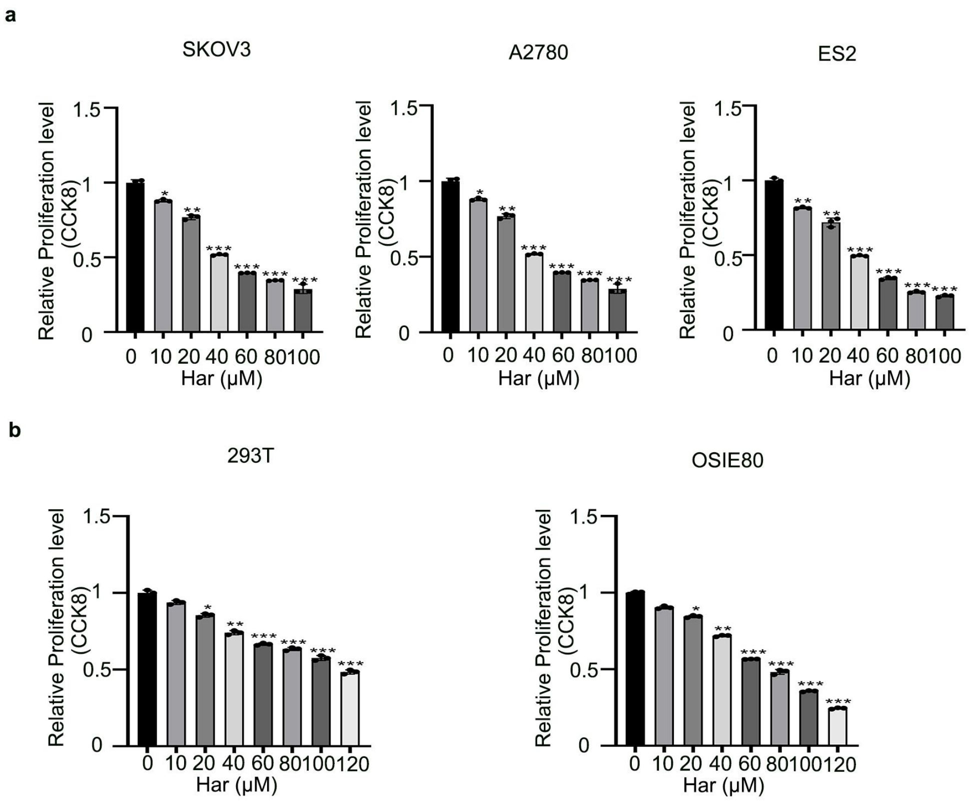


**Fig S0 Efficacy and dose safety of Har in inhibiting ovarian cancer**

(a) Dose-dependent effects of Har on the proliferation of SKOV3, A2780, and ES2 cells. (b) Dose-dependent effects of Har on the proliferation of non-cancer cell lines 293T, ISOE80 cells. All results are expressed as the mean±SD. * $p < 0.05$, ** $p < 0.01$, *** $p < 0.001$ vs. the control group. Har, Harmine.
