# Supplementary figures and images for: The roles of autophagy, ferroptosis and pyroptosis in the anti-ovarian cancer mechanism of harmine and their crosstalk
Source: Sci Rep. 2024 Mar 18;14:6504. doi: 10.1038/s41598-024-57196-7 (PMC10948856; doi:10.1038/s41598-024-57196-7)

Fig S1d

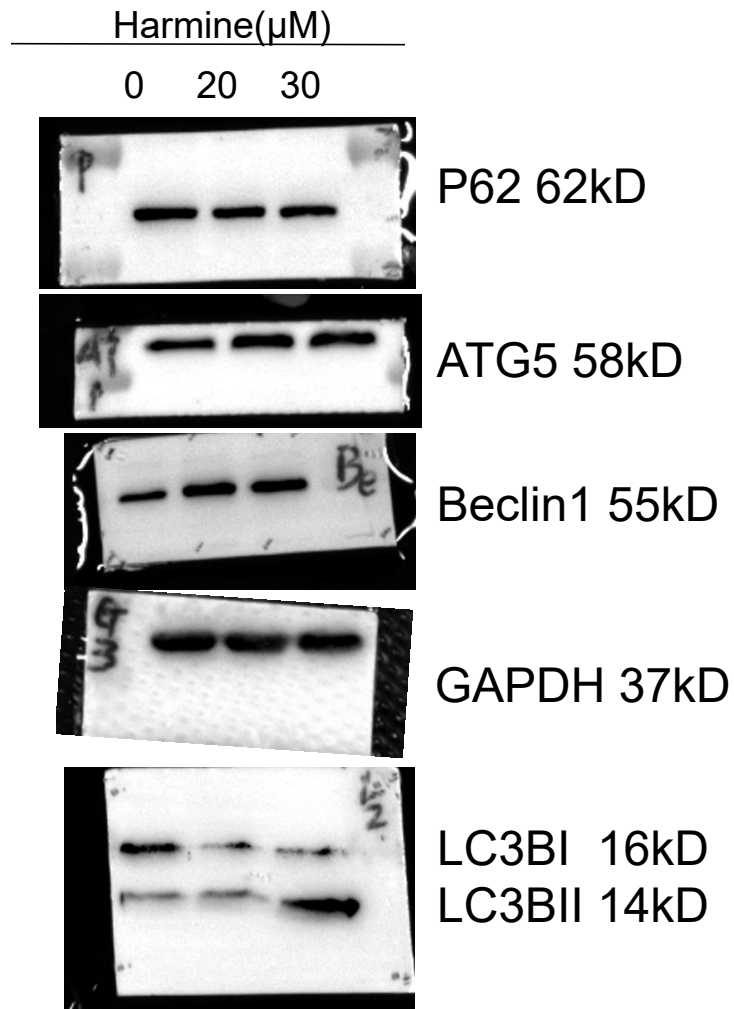

Supplement: Supplementary file 2 — Supplementary Information 2. [file 41598_2024_57196_MOESM2_ESM.pdf]

Fig S1e

|       |   |   |   |   |
|-------|---|---|---|---|
| Har   | - | + | - | + |
| BafA1 | - | - | + | + |

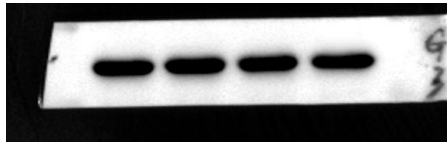

GAPDH 37kD

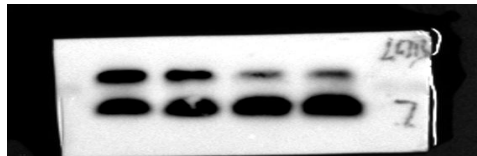

LC3BI 16kD  
LC3BII 14kD

|      |   |   |   |   |
|------|---|---|---|---|
| Har  | - | + | - | + |
| 3-MA | - | - | + | + |

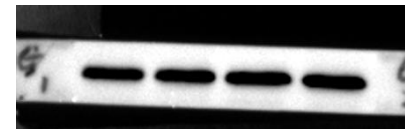

GAPDH 37kD

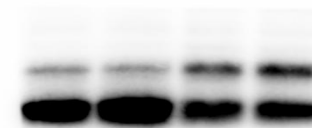

LC3BI 16kD  
LC3BII 14kD

Supplement: Supplementary file 3 — Supplementary Information 3. [file 41598_2024_57196_MOESM3_ESM.pdf]

Fig S2a

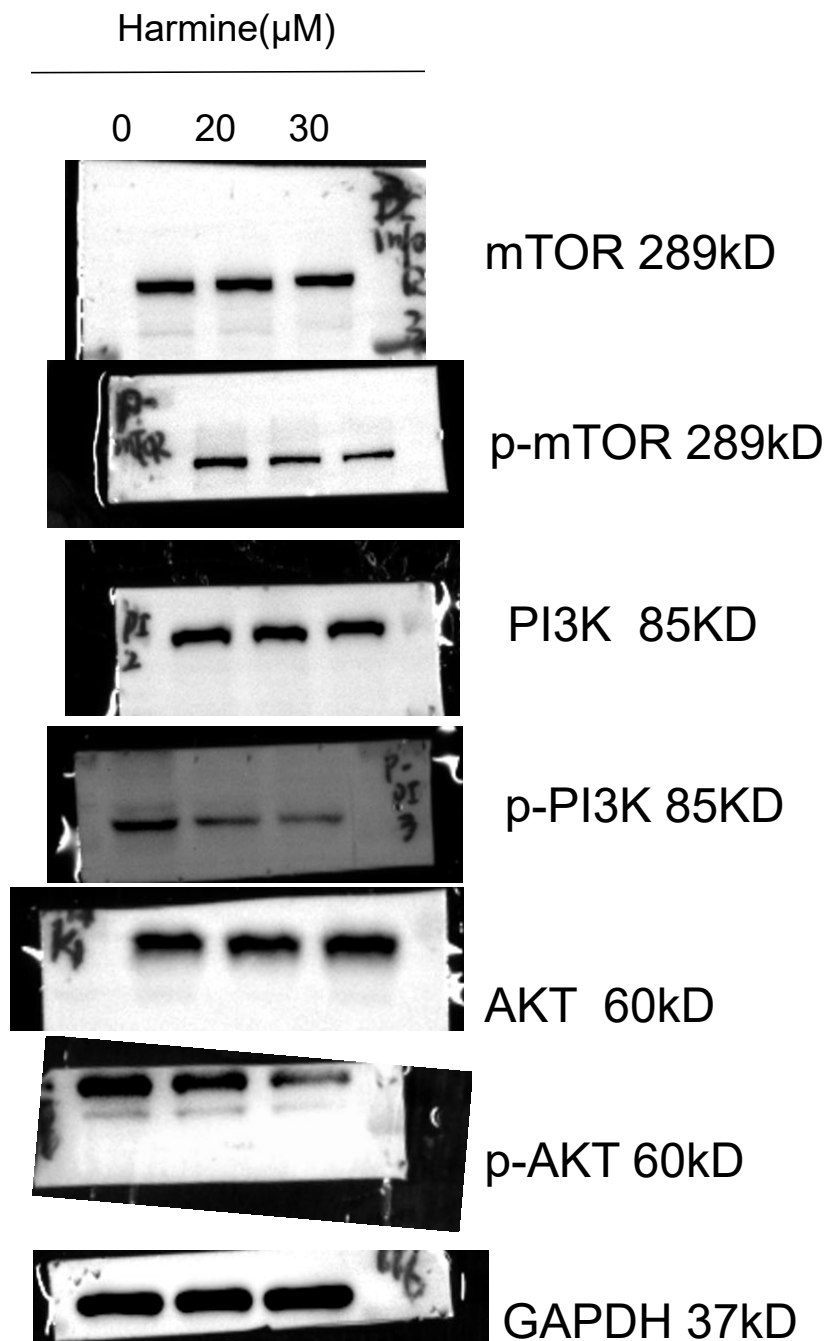

Supplement: Supplementary file 4 — Supplementary Information 4. [file 41598_2024_57196_MOESM4_ESM.pdf]

Fig S2b

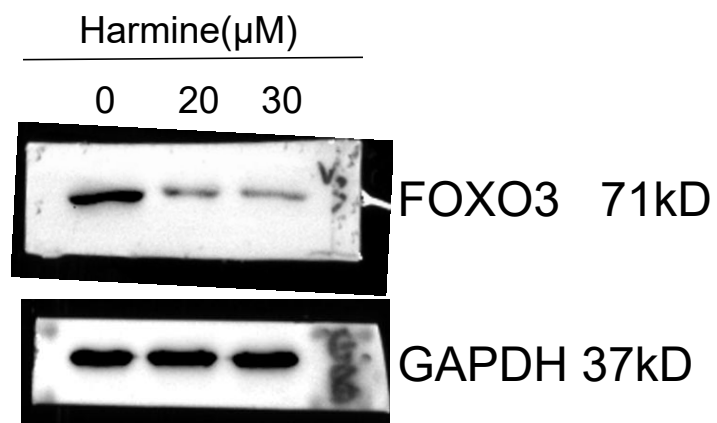

Supplement: Supplementary file 5 — Supplementary Information 5. [file 41598_2024_57196_MOESM5_ESM.pdf]

Fig S2c

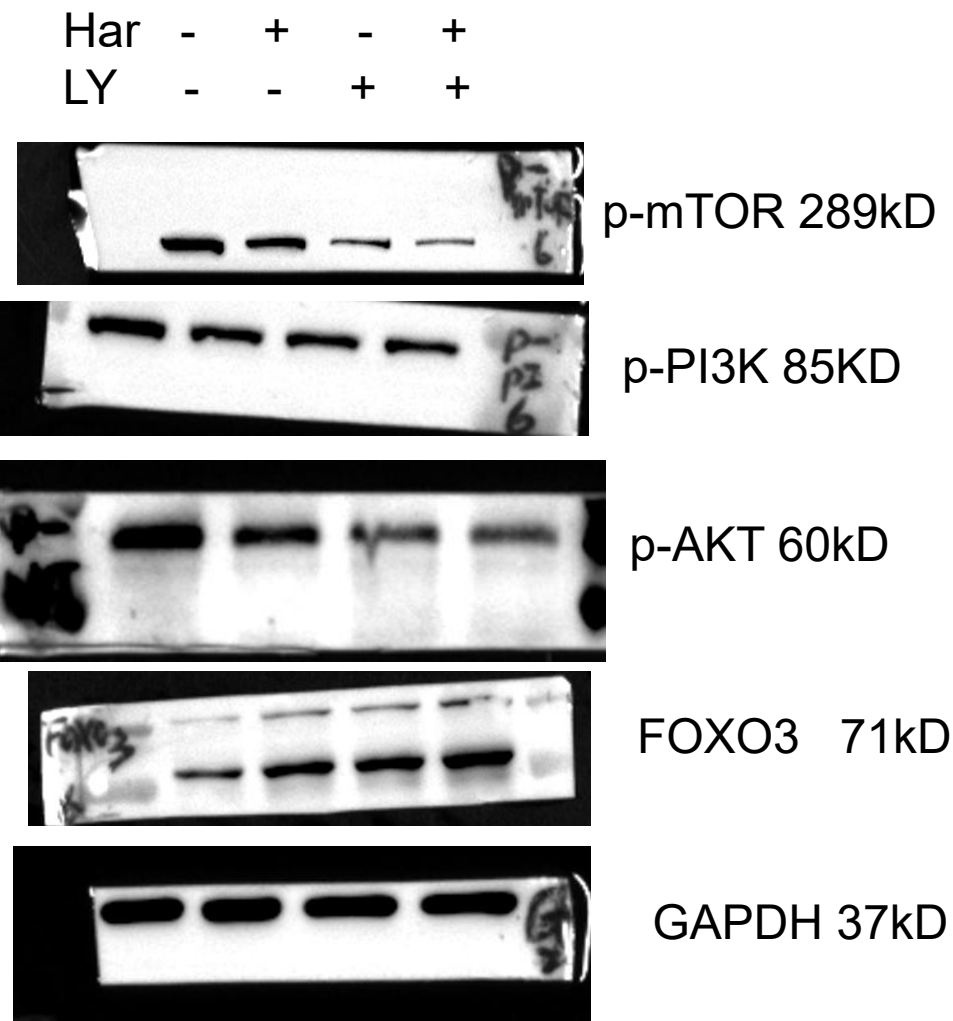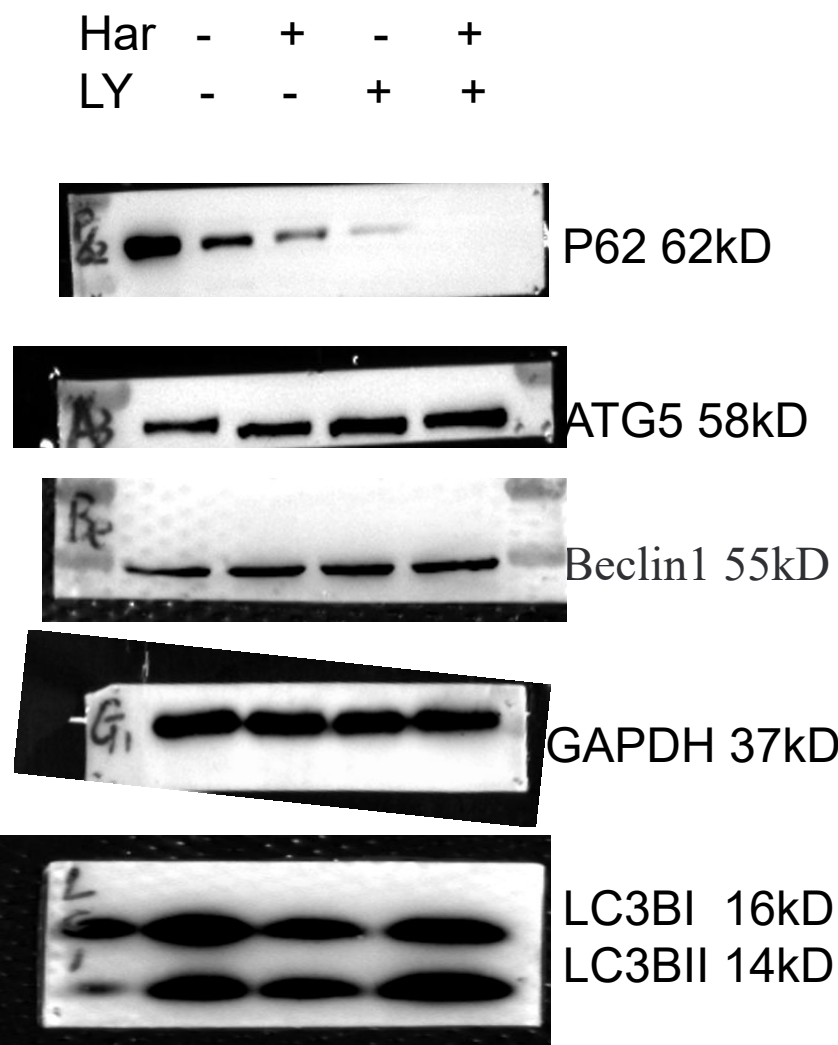

Supplement: Supplementary file 6 — Supplementary Information 6. [file 41598_2024_57196_MOESM6_ESM.pdf]

Fig S2d

|            |   |   |   |   |
|------------|---|---|---|---|
| si-FOXO3-1 | - | + | - | + |
| si-FOXO3-2 | - | - | + | + |

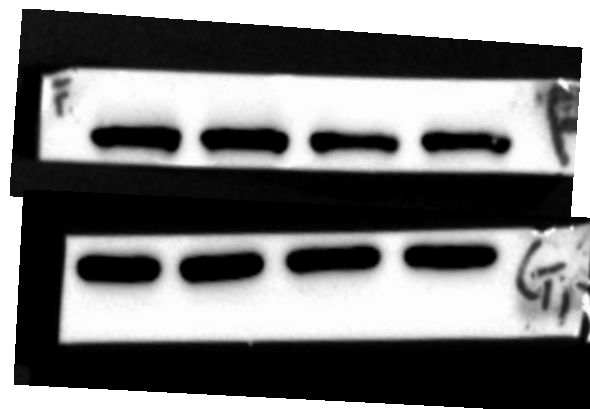

FOXO3 71kD

GAPDH 37kD

Supplement: Supplementary file 7 — Supplementary Information 7. [file 41598_2024_57196_MOESM7_ESM.pdf]

Fig S2e

|            |   |   |   |   |   |   |
|------------|---|---|---|---|---|---|
| Har        | - | + | - | + | - | + |
| si-FOXO3-1 | - | - | + | + | - | - |
| si-FOXO3-2 | - | - | - | - | + | + |

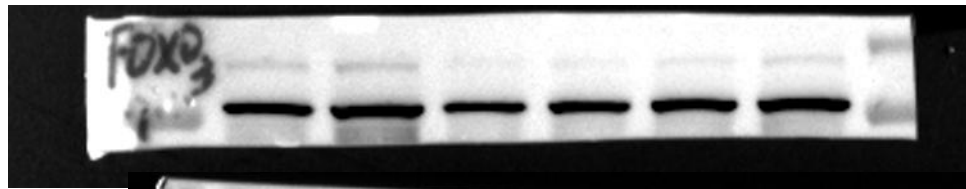

FOXO3 71kD

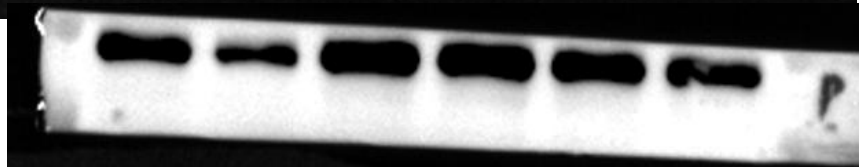

P62 62kD

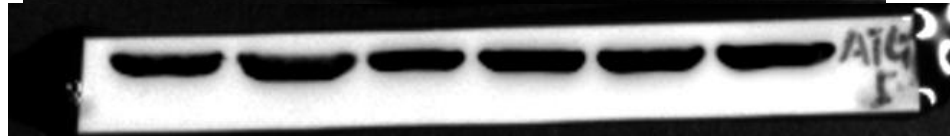

ATG5 58kD

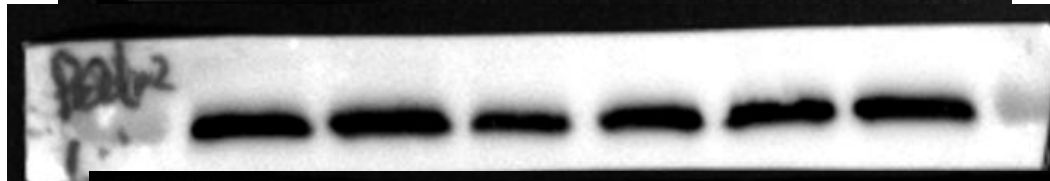

Beclin1 55kD

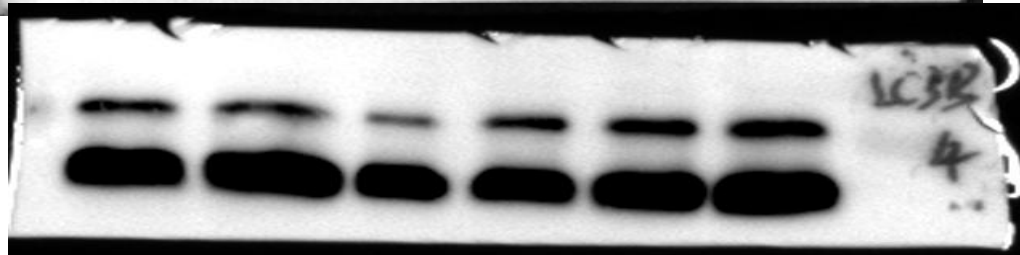

GAPDH 37kD

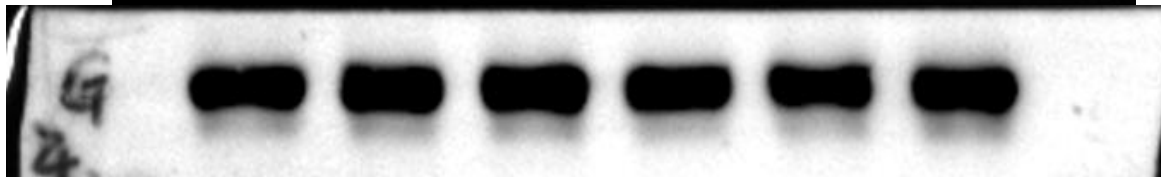

LC3BI 16kD  
LC3BII 14kD

Supplement: Supplementary file 8 — Supplementary Information 8. [file 41598_2024_57196_MOESM8_ESM.pdf]

Fig S3b

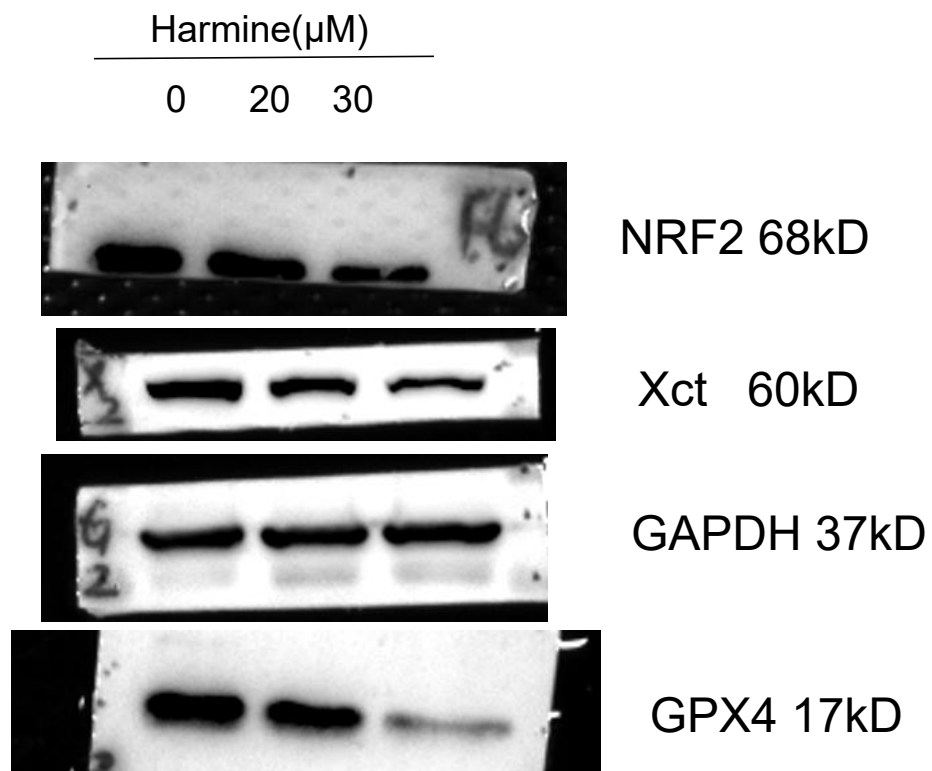

Supplement: Supplementary file 9 — Supplementary Information 9. [file 41598_2024_57196_MOESM9_ESM.pdf]

Fig S3c

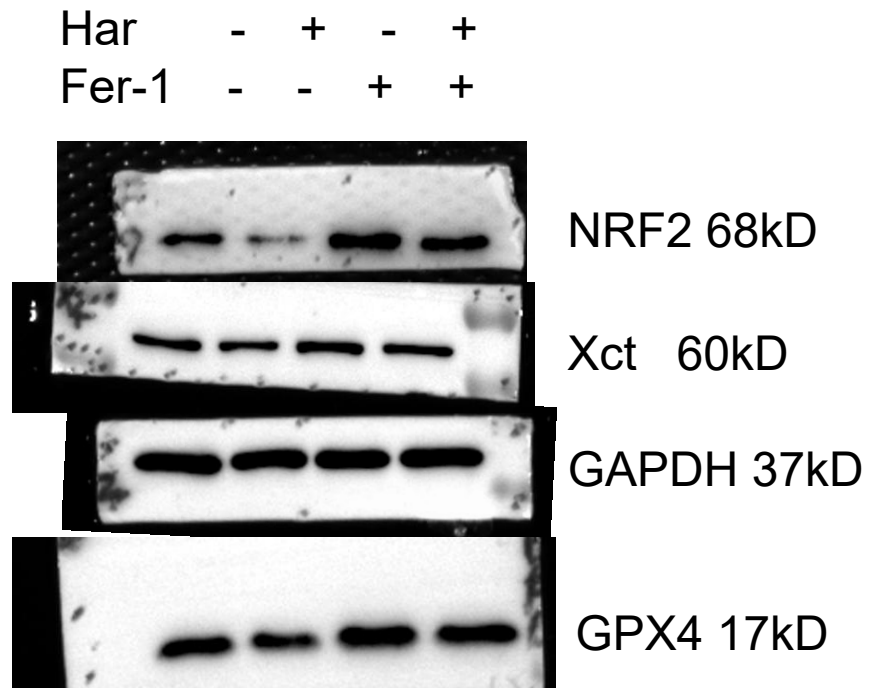

Supplement: Supplementary file 10 — Supplementary Information 10. [file 41598_2024_57196_MOESM10_ESM.pdf]

Fig S4a

|     |   |   |   |   |
|-----|---|---|---|---|
| Har | - | + | - | + |
| Rap | - | - | + | + |

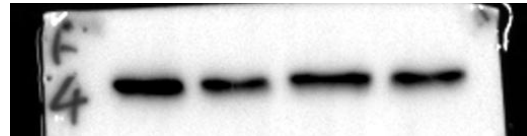

NRF2 68kD

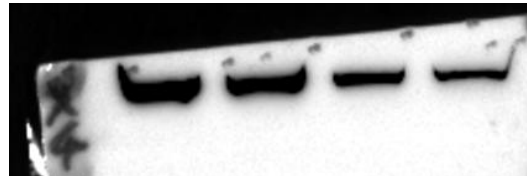

Xct 60kD

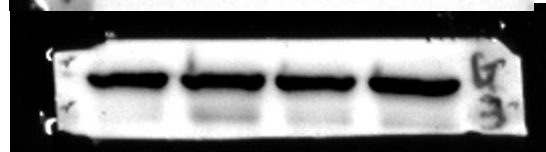

GAPDH 37kD

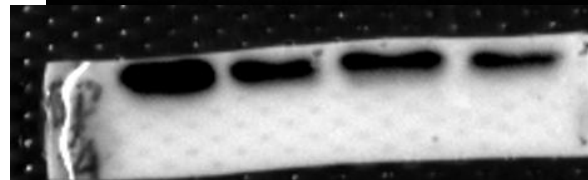

GPX4 17kD

Supplement: Supplementary file 11 — Supplementary Information 11. [file 41598_2024_57196_MOESM11_ESM.pdf]

Fig S4b

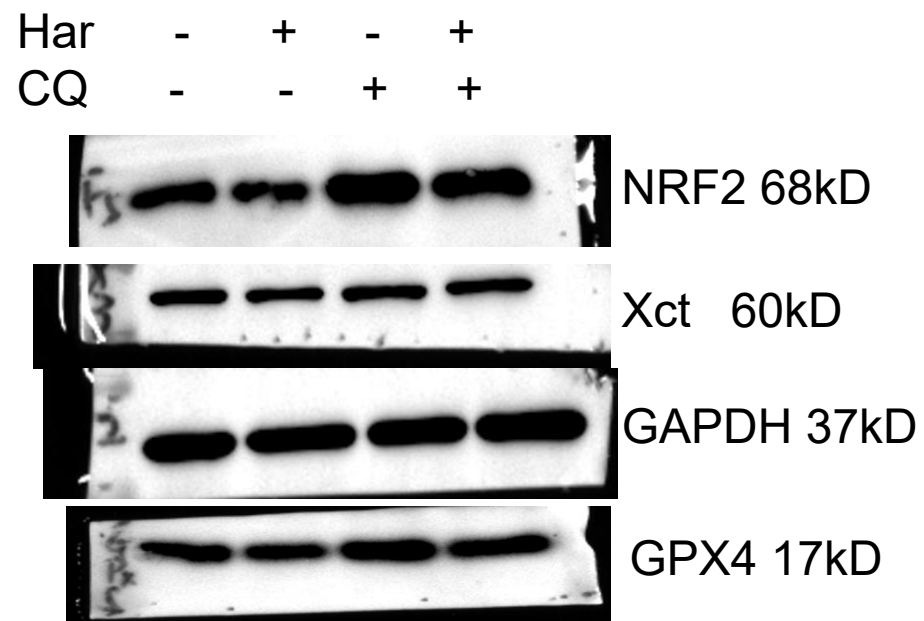

Supplement: Supplementary file 12 — Supplementary Information 12. [file 41598_2024_57196_MOESM12_ESM.pdf]

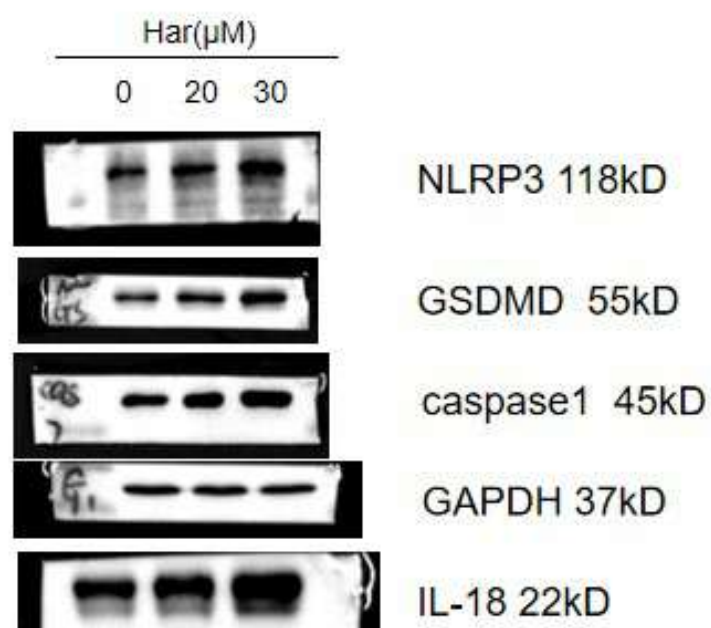

Supplement: Supplementary file 13 — Supplementary Information 13. [file 41598_2024_57196_MOESM13_ESM.pdf]

CQ( $\mu$ M)

| 0 | 30 | 50 |
|---|----|----|
|---|----|----|

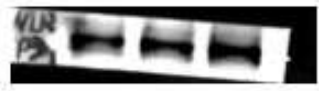

NLRP3 118kD

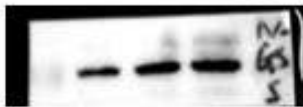

GSDMD 55kD

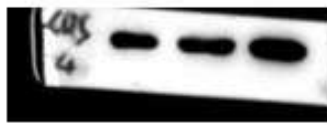

caspase1 45kD

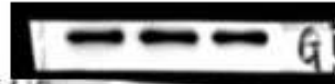

GAPDH 37kD

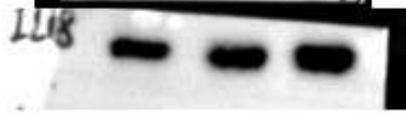

IL-18 22kD

Rap(nM)

| 0 | 200 | 400 |
|---|-----|-----|
|---|-----|-----|

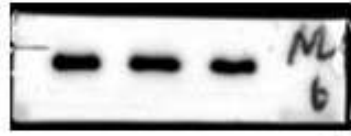

NLRP3 118kD

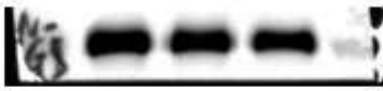

GSDMD 55kD

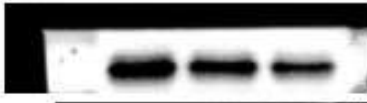

caspase1 45kD

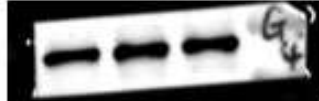

GAPDH 37kD

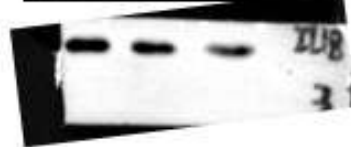

IL-18 22kD

Supplement: Supplementary file 14 — Supplementary Information 14. [file 41598_2024_57196_MOESM14_ESM.pdf]

|     |   |   |   |   |
|-----|---|---|---|---|
| Har | - | + | - | + |
| CQ  | - | - | + | + |

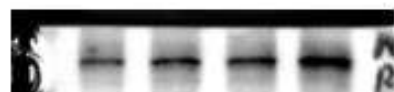

NLRP3 118kD

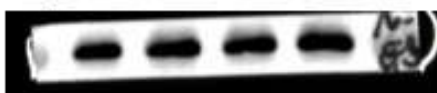

GSDMD 55kD

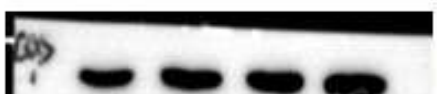

caspase1 45kD

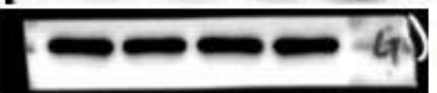

GAPDH 37kD

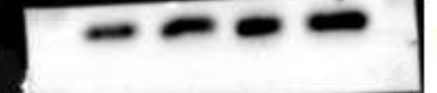

IL-18 22kD

|     |   |   |   |   |
|-----|---|---|---|---|
| Har | - | + | - | + |
| Rap | - | - | + | + |

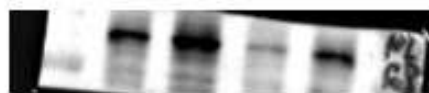

NLRP3 118kD

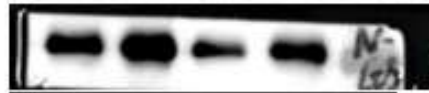

GSDMD 55kD

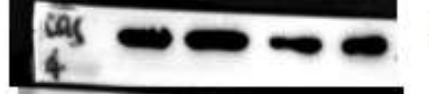

caspase1 45kD

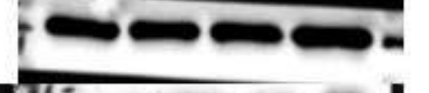

GAPDH 37kD

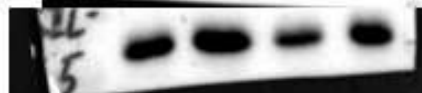

IL-18 22kD

Supplement: Supplementary file 16 — Supplementary Information 16. [file 41598_2024_57196_MOESM16_ESM.pdf]
