# Supplementary material for: The roles of autophagy, ferroptosis and pyroptosis in the anti-ovarian cancer mechanism of harmine and their crosstalk
Source: Sci Rep. 2024 Mar 18;14:6504. doi: 10.1038/s41598-024-57196-7 (PMC10948856; doi:10.1038/s41598-024-57196-7)
Supplement: Supplementary file 17 — Supplementary Information 17. [file 41598_2024_57196_MOESM17_ESM.pdf]

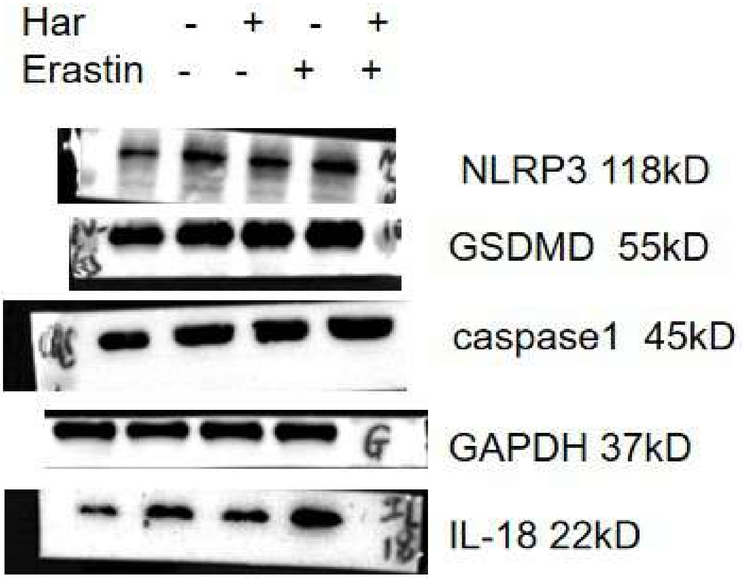
Har - + - +
Erastin - - + +
NLRP3 118kD
GSDMD 55kD
caspase1 45kD
GAPDH 37kD
IL-18 22kD

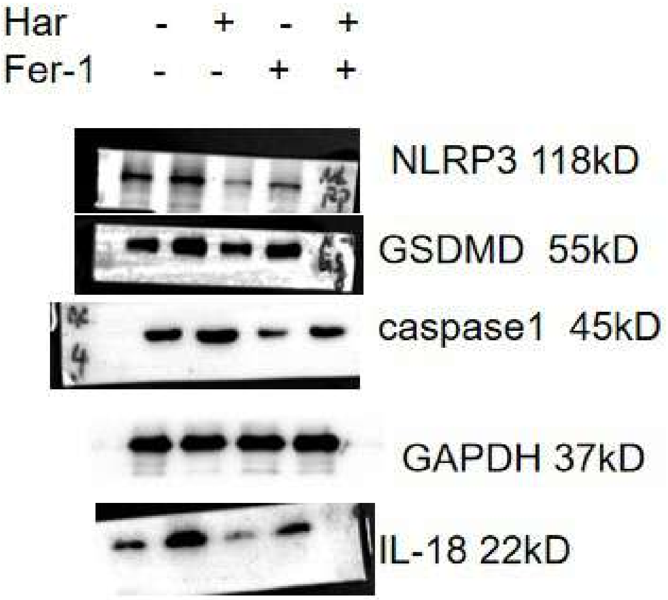
Har - + - +
Fer-1 - - + +
NLRP3 118kD
GSDMD 55kD
caspase1 45kD
GAPDH 37kD
IL-18 22kD
